# Extrapolating potential crop damage by insect pests based on land use data: examining inter-regional generality in agricultural landscapes

**DOI:** 10.1186/s12862-022-02024-7

**Published:** 2022-05-25

**Authors:** Ken Tabuchi, Akihiko Takahashi, Ryuji Uesugi, Shigeru Okudera, Hideto Yoshimura

**Affiliations:** 1 Tohoku Agricultural Research Center, NARO, 4 Akahira, Shimo-kuriyagawa, Morioka, Iwate 020-0198 Japan; 2 Hokuriku Research Station, Central Region Agricultural Research Center, NARO, 1-2-1 Inada, Joetsu, Niigata 943-0193 Japan; 3 Laboratory of Biology, Asahikawa Campus, Hokkaido University of Education, 9 Hokumon-cho, Asahikawa, 070-8621 Japan

**Keywords:** Area-wide pest management, Pecky rice, Pest-suppressing landscape, *Stenotus rubrovittatus*, Landscape structure

## Abstract

**Background:**

Inter-regional relationships between landscape factors and biological responses in natural conditions are important but difficult to predict because of the differences in each landscape context and local environment. To examine the inter-regional variability in relation to landscape factors and the biological response of an insect pest of rice, *Stenotus rubrovittatus*, we extrapolated a damage prediction model (the ‘original model’ of our previous study) for rice using land-use data. The ‘original model’ comprised as fixed factors the area of source habitat (i.e. pastures and graminoid-dominated fallow fields), soybean fields, and rice paddies within 300-m radii with research years as the random intercept. We hypothesized that the original model would be applicable to new regions, but the predictive accuracy would be reduced. We predicted that fitting a new extended model, adjusting the parameter coefficients of identical fixed factors of the ‘original model,’ and adding regional random intercepts would improve model performance (the ‘extended model’). A field experiment was conducted in two regions that had a similar landscape context with the original region, each in a different year of four years in total. The proportion of rice damage and surrounding land use within a 300-m radius was investigated, and the data were applied to the models and the applicability and accuracy of the models were examined.

**Results:**

When the ‘original model’ was assigned to the combined data from the original and extrapolated regions, the relationship between the observed and the predicted values was statistically significant, suggesting that there was an inter-regional common relationship. The relationship was not statistically significant if the model was applied only to the new regions. The extended model accuracy improved by 14% compared with the original model and was applicable for unknown data within the examined regions as demonstrated by three-fold cross validation.

**Conclusions:**

These results imply that in this pest–crop system, there is likely to be a common inter-regional biological response of arthropods because of landscape factors, although we need to consider local environmental factors. We should be able to apply such relationships to identify or prevent pest hazards by offering region-wide management options.

**Supplementary Information:**

The online version contains supplementary material available at 10.1186/s12862-022-02024-7.

## Background

Evaluation of the response of organisms to landscape factors is important for solving basic and applied problems, such as species conservation, prediction of the population dynamics of organisms, and pest management [1–3]. The temporal and spatial effects of landscape factors on the biological responses of organisms vary from region to region (e.g. [4]). Previous studies have reported that the relationships between landscape factors and biological responses depend on dispersal ability, local and inter-habitat movement, population density, habitat related contexts (i.e. size, amount, heterogeneity, and fragmentation), and associated responses of organisms [3]. Inter-regional variations in landscape context, that is, the amount and spatial pattern of different land cover types surrounding a given site [5], is expected to cause changes in these relationships. However, there has been very little examination of the extent of inter-regional variation in landscape factors, such as land use composition and configuration, and biological responses in similar environments.

Addressing this issue will be valuable in terms of applied ecology, because it offers the prospect of being able to extrapolate environmental hazards, such as the likelihood of pest damage, wildlife and vector-borne diseases, and their management actions. Compared with natural and semi-natural landscapes which are highly diverse and contextually unique, agricultural landscapes, which cover 37% of the global land area [6], have relatively simple plant and animal communities. Conventionally managed fields are particularly environmentally homogenous because of the management style of local farmers in terms of mowing, cultivation, and agrochemical use. In agricultural landscapes, especially in intensively managed agricultural environments, comparable responses of organisms to landscape factors may be observed even in different regions because of the similarity of anthropogenic management. Discussions about extrapolation have been a major research focus in applied ecology [4]. However, few studies of species occurrence have discussed commonality across regions [7, 8], and more complex responses such as crop damage have not been tested using empirical data. Knowing whether there are inter-regional generalities in local ecosystem management that can be applied regionally may help us to develop recommendations for stakeholders for nature conservation and biological management policies.

*Stenotus rubrovittatus* is one of the most important rice pests in eastern Asian countries including Japan and South Korea [9, 10]. A predictive model of rice damage from this insect (known as “pecky rice damage” on brown rice grains), using land use within an effective spatial scale was developed, and a hazard map was prepared based on the model [11]. This species seldom breeds in rice paddies [12] and the number of individuals of *S. rubrovittatus* invading rice paddy fields is limited by the source habitat of the surrounding landscape. This model predicts rice damage in the focal field using land use data within a 300-m radius, mainly based on the area of source habitat (i.e. pastures and graminoid-dominated fallow fields). Similar results on the effective spatial scale of *S. rubrovittatus* abundance were reported in two independent regions, which are 300 km away from each other, suggesting some inter-regional generality [13, 14]. The damage prediction model and the hazard map for *S. rubrovittatus* can be applied to improve management options for the pest and to allocate the optimal labor required for spraying pesticides. It is anticipated that the performance of the predictive model will improve as it becomes available in wider areas. Developing a damage prediction model for pests in a single region that can be applicable to neighboring regions may help to predict pest damage in wider areas, and further enhance the development of effective pest management practices.

To examine whether the inter-regional generality of the relationship between landscape factors and crop damage could be established in agricultural landscapes that have similar contexts, a field study was carried out in two extra regions that had a similar landscape context with the original region, each in a different year of 4 years in total. The data obtained in the two regions were compared with that of the original region of 3 years [11]. We extrapolated a damage prediction model to two different areas that had similar landscape contexts to the region where the damage prediction model was developed. We hypothesized that the relationship between landscape factors and crop damage would be comparable across regions with similar landscape contexts, and that it would be possible to extrapolate the damage prediction model. We also predicted that the accuracy of the model’s prediction would be reduced in extrapolated regions presumably owing to the influence of regional and local factors, such as pest abundance variation and associated factors relating to local land-use patterns, necessitating adjustments in parameter coefficients.

## Results

**Fig. 1:**
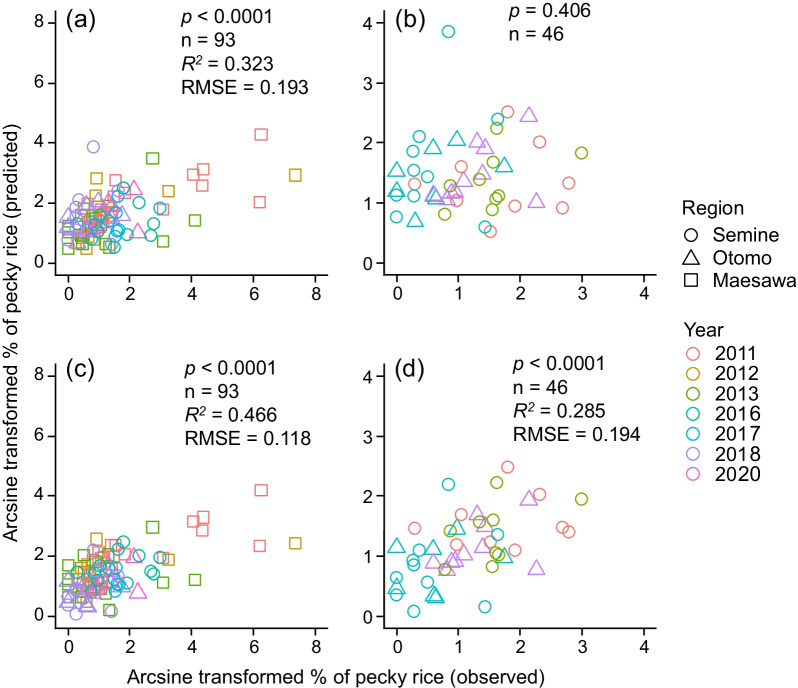
Post hoc comparison of the relationship between the observed and predicted values (%) for pecky rice: the original model **a** for the combined data; and **b** the data of the current study; and the extended model **c** for the combined data; and **d** the data of current study. All data were calculated after arcsine transformation. Reported *R*^2^ values are the conditional *R*^2^, which were estimated for the mixed models including the random effects

When we checked the parallel slopes of regression lines to combine the data from two new regions with 4 years and one previous region with 3 research years, the model indicated that no significant regional difference was observed (LM: *F* = 0.091, df = 1, *p* = 0.789, Additional file 1: Table S2). In addition, no significant difference was observed for the two-way interactions between each land use and region (LM: *p* > 0.05, Additional file 1: Table S2 and Figs. S1 and S2) on the arcsine-transformed percentage of pecky rice damage. These results supported the existence of an inter-regional common pattern among the pecky rice damage and the area of land uses. However, only the two-way interaction of source habitat and year was significant (LM: *F* = 15.21, df = 5, *p* = 0.047, Additional file 1: Table S2), indicating that the research year had a significant effect on the coefficient between pecky rice damage and the area of source habitat. For the combined data of the current study and that of the previous study [11], which contained 93 samples, the comparison of observed and predicted values for pecky rice damage by the original predictive model was statistically significant (Fig. 1a), indicating that an inter-regionally common pattern was observed. However, when only the data for the two current study regions was assigned to the original predictive model of pecky rice damage, the relationship varied and was not statistically significant (LMM: *n* = 46, *χ*^2^ = 0.705, *p* = 0.401; Fig. 1b). The area under the receiver operating characteristic curve (AUC) value was 0.50 (Table 1), indicating the model performance was poor and showed almost no discrimination ability to the current dataset. However, the discrimination accuracy of the model exceeded 50% and was 67.4%, despite the small range in pecky rice damage (0% to 0.27%).

**Table 1:** Comparison of the model performance between the original and extended predictive models for the combined data and current study data

Data	Model	*R*^*2*^	RMSE	Accuracy (%)	AUC	Sensitivity	Specificity	Positive predictive value	Negative predictive value
Combined	Original	0.323	0.157	72.0	0.73	61	75	37	89
(*n* = 93)	Extended	0.466	0.118	77.4	0.79	67	80	44	91
Current study	Original	–^a^	–^a^	67.4	0.50	33	73	15	88
(*n* = 46)	Extended	0.285	0.193	82.6	0.73	33	90	33	90

^a^The model was not significant (LMM, *t* = 0.839, *p* > 0.05)

For the 93 samples of the combined data from the current study and Tabuchi et al. [11], we created an extended predictive model of rice damage, for which the model equation is shown as below:

1 $$y = \, 0.0{3 } + { 32}.0{7}*{\text{Source habitat }} + { 44}.{5}*{\text{Soybean }} + { 2}.{6}*{\text{Paddy field }} + {\text{ RI}}_{{{\text{region }}\& {\text{ year}}}}$$

2 $${\text{RI}}_{{{\text{region }}\& {\text{ year}}}} = u_{{{\text{region}}}} + w_{{\text{year in region}}}$$

where y is the arcsine-transformed pecky rice damage at given points, 0.03 is the fixed intercept of the model, and the areas of source habitat, soybean fields, and paddy fields were calculated within 300-m radii of the research points (km^2^). These were the fixed part of the model. The RI_region & year_ was the random intercept (Eq. 2), which consisted of region-specific intercepts (*u*_region_) and year-specific random intercepts in each region (*w*_year in region_). All parameters of the original model were selected based on the Akaike information criterion (AIC)-based best model (Table 2), indicating that there was an inter-regional common relationship between landscape factors and rice damage. In the extended model, the coefficients for source habitat and soybean fields slightly decreased by 7.98 point and 8.2 point, respectively (Additional file 1: Table S2). The second-best model consisted of the area of source habitat and soybean fields (Table 2).

**Table 2:** Summary of the top five linear mixed models describing the variation in the prevalence of pecky rice with land use within a 300-m radius from each research point

ΔAICc	Weight	df	Source habitat	Soybean field	Paddy field	Elevation	Temperature				Intercept
							May	June	July	August	
–	0.306	7	32.07***	44.52*	2.61	–	–	–	–	–	0.03
1.63	0.135	6	32.20***	44.26*	–	–	–	–	–	–	0.28
2.52	0.087	8	33.04***	45.70*	2.49	–	0.17	–	–	–	–2.52
2.72	0.078	8	32.40***	44.65*	2.74	–	–	–	–0.04	–	0.73
3.27	0.059	8	32.48***	44.78*	2.68	–	–	–	–	–0.07	1.64

The values of land use indicate model coefficients

Each value of the model was calculated with the function “lme ()” of the nlme package in R

Bold characters indicate significant variables

Temperature in September was not included owing to high multicollinearity with that of August

The variation inflation factors for all models showed no multicollinearity

^†^< 0.10, *< 0.05, **< 0.01, ***< 0.001. Values without * or † are not significant

By comparing the relationship between the observed and predicted values of pecky rice damage, the *R*^2^ value of the extended model improved by 14% (Fig. 1a and c) over the *R*^2^ value of the original model [11]. The predicted values of lower damage seemed relatively stable and were improved for the extended model with the current study data (Fig. 1d); however, the predicted higher damage range was underestimated, and the tendency for underestimation did not improve with the combined data (Fig. 1c). The AUC value improved from 0.73 for the original model to 0.79 for the extended model, indicating that both models had moderate accuracy (Table 1). When comparing the sensitivity, specificity, and positive and negative predictive values of the original model to the combined data, the specificity and positive predictive values were improved by the extended model.

The relationship between the observed and predicted values of pecky rice damage from the current study data was statistically significant in the extended model (LMM: *n* = 46, *χ*^2^ = 17.30, *p* < 0.001; Fig. 1d). The AUC value of the extended model for only the data of the current study was improved to 0.74 (Table 1). In the extended model, the specificity and positive predictive values were improved, indicating that the extended model was more accurate for specificity and predicted true positives (Table 1). From the three-fold cross validation, which tested the model’s accuracy for unknown data within three regions, the *R*^2^ value between the observed and predicted pecky rice damage was 0.50 ± 0.09 (mean ± SD), and the mean AUC value ± SD was 0.73 ± 0.13, indicating that model accuracy for unknown data was moderate.

**Fig. 2:**
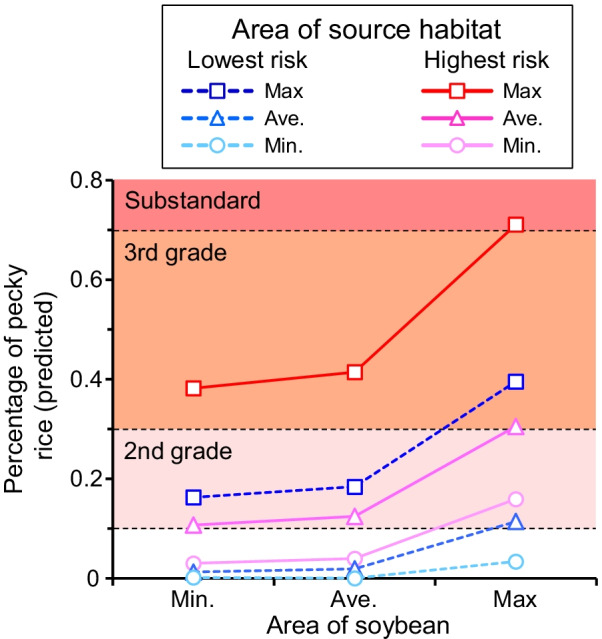
Percentage of predicted pecky rice damage fitted by the extended model that represents the sensitivity of the model prediction. The lowest (Semine region in 2018, blue dotted lines) and highest (Maesawa region in 2011, red and pink solid lines) risk cases are demonstrated. The fitted values were calculated using the minimum, average, and maximum areas of source habitat (0.37, 3.12, and 8.32 ha, respectively) and soybean fields (0, 0.33, 2.90 ha, respectively), and the average area of rice paddy fields (9.42 ha). Circles, triangles, and squares indicate the minimum, average, and maximum areas of source habitat. The background color of the graph indicates the grades of pecky rice damage

The predicted values of pecky rice damage in the extended model were higher with increase in the area of source habitat and soybean fields (Fig. 2). Regardless of the lowest (Semine region in 2018) and the highest (Maesawa region in 2011) risk cases, the rice grade decreased to second grade or lower in the case of the maximum area of source habitat, indicating that the risk of possible price decline is high under this situation.

**Fig. 3:**
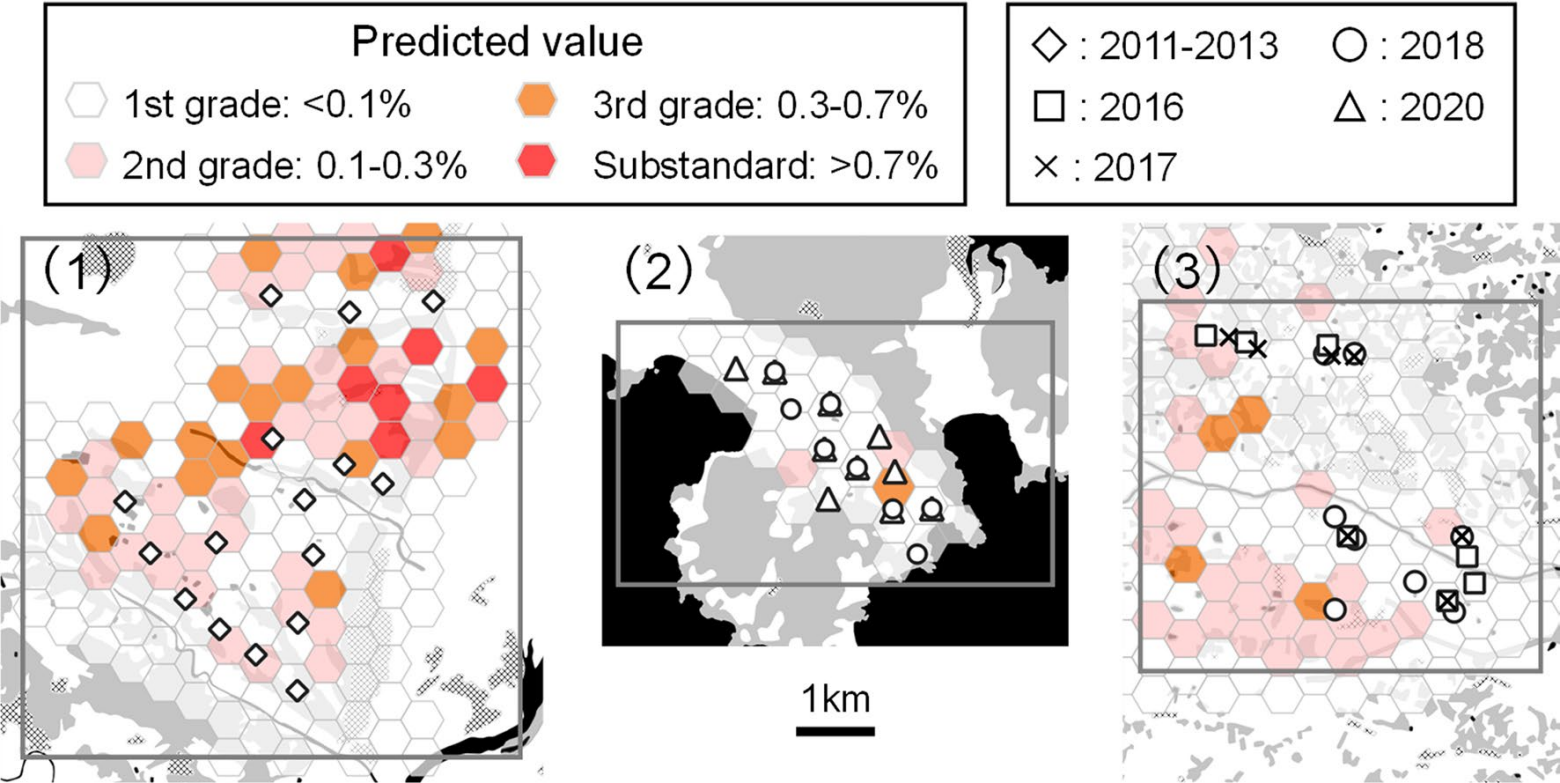
Priority area map of potential pecky rice damage in agricultural fields with a grid layer of 300-m hexagons. (1) Maesawa region, Oshu city, (2) Otomo region, Rikuzen-Takata city, and (3) Semine region, Kurihara city. Gray, black, and dotted areas represent forest, open water, and other land uses, respectively. Shapes in the figure indicate the studied rice paddy fields in each research year (see also Fig. 4). Squares indicate regions examined. Predicted values in hexagons were calculated using the land-use data in 2013 of Maesawa, 2018 of Otomo, and 2016 of Semine region. The model prediction of the map does not contain any region- and year-specific effects

The potential priority areas were mapped using the model (Fig. 3). The 95 priority areas were selected from among 380 hexagons. Thus, the priority areas for pest management were successfully visualized. The model prediction varied between the highest- and the lowest-risk cases, and 167 and 49 priority areas were selected, respectively (Additional file 1: Fig. S3).

## Discussion

Our study supported the hypothesis that an inter-regional common relationship was observed between landscape factors and crop damage by a pest across three agricultural landscapes. The original predictive model was applicable to all regions within the current study and the original region (Fig. 1a, Table 2), suggesting that the original model is able to predict rice damage with moderate accuracy. However, direct extrapolation of the original damage prediction model to only the extrapolated regions of the current study was not straightforward (Fig. 1b, Table 2), resulting in lower accuracy than in the original region of the model. Data from the current study did not include a degree of damage higher than four for the arcsine-transformed pecky rice damage, thus, model accuracy may vary in the region or year of higher risk of pecky rice damage. Previous studies dealing with both empirical and theoretical arguments suggest that effective spatial scale and associated biological responses against landscape factors would differ between regions even with the same species (reviewed in [3]) and, thus, extrapolation would not be easy. The current study examined identical species using the same spatial scale in several research regions. We empirically demonstrated that the effective spatial scale of the pest was inter-regionally applicable in relation to landscape factors and crop damage by *S. rubrovittatus*, although other spatial scales were not examined.

Our results showed that adjustment of the parameter coefficients of the predictive model by adding additional regional data can improve predictions when we use models developed over a wider area. The relationships between landscape factors and responses of different arthropod pests are highly dependent on the pest taxa and the nature of their trophic interactions (e.g. [15]). The trophic interaction of our study between *S. rubrovittatus* and its natural enemies is relatively simple compared with previous studies. Irrigated rice fields are the most biologically diverse agroecosystems, and regional and local conditions affect rice arthropod communities [16, 17]. However, insecticides also reduce species diversity and abundance in rice agroecosystems [18–20]. Top-down regulation of *S. rubrovittatus* is not strongly expected in this study, because the population of such natural enemies is suppressed by conventional management, such as spraying insecticides [21–23] and the species has almost no specific natural enemies other than spiders [14]. *Stenotus rubrovittatus* seldom reproduces in rice paddy fields [10], so the number of individuals invading rice paddy fields is mostly determined by the source habitat of the surrounding landscape. Therefore, our study may not be directly applicable to other pests, crops, and agricultural landscapes. However, our results suggest that inter-regional commonality of biological responses to landscape factors may exist at some scales in the population dynamics of pests and organisms that have similar ecological characteristics with *S. rubrovittatus*.

In this study, there were several common landscape factors. For example, we carefully selected regions with rice-dominated landscapes and similar major crop composition, rice growing season, and management intensity for insect pests (i.e. the number of pesticide applications). In addition, the local environment and management intensity of pests and weeds in each field were very similar, and the species composition of major insect pests was similar. In contrast, several factors, such as the research year and associated climatic conditions, and the quality of source habitat of the pest (i.e. species composition of graminoid weeds) differed between the regions examined. Furthermore, the percentage of *S. rubrovittatus* among major grain-attacking pest species in the regions varied. These factors might change the accuracy of the original predictive model of crop damage. However, the extended predictive model here could be assigned within 50 km^2^ in this study; similar biological responses of planthopper pests were shown at an equivalent spatial scale in the extra fine-grained Asian rice landscapes in China [24]. Moreover, it was possible to extrapolate the predictive model over wider areas that had similar environmental conditions. For example, similar results on the relationships between *S. rubrovittatus* abundance and landscape factors at similar spatial scales were reported in two independent regions, which were 300 km apart [13, 14]. Over large areas of hundreds of kilometers, previous studies have shown common patterns in the response of landscape factors and biological responses of arthropods [25–27], although there has been no attempt to extrapolate using empirical validation as done in the current study. Therefore, the extended predictive model may be able to extrapolate even further from the area of the current study if the environmental and biological context is similar.

Further improvement of the accuracy and sensitivity of the predictive model should be considered, however. The extended model prediction varied among the lowest and highest risk cases (Fig. 3, Additional file 1: Fig. S3), indicating that regional and other related factors, which were not considered in this study, affected the model fitting. The prediction accuracy of our extended predictive model was demonstrated to be moderate, and the accuracy of the model might be improved by incorporating such factors. Our predictive model only included land use; therefore, incorporating values relating to management practices or pre-cropping climatic conditions and other unknown factors may improve the model’s accuracy in the future. A combination of land use, climatic conditions, and pest management practices affects population dynamics in cotton pests [27] and soybean aphids [26], which is reasonable to explain the population dynamics of poikilothermic organisms. Previous studies of rice-attacking pentatomids in Japan suggested that the temperature of the rice heading period and grain-filling period has a positive effect on the total pecky rice damage of several pentatomid species, including *S. rubrovittatus* in southwestern Japan [28] and the abundance of another mirid species, *T. caelestialium*, in northern Japan [29]. However, elevation and temperatures were not statistically significant beyond regions, indicating that these factors did not affect the predictive model in this study. Our study included only three regions, and research fields were clustered in each region, which may have resulted in less variability in climatic conditions. Therefore, the effect of temperature might not be detected in the current data set, and adding more regions, temperature, and other climatic conditions might improve the predictive model as factors potentially explaining pecky rice damage.

Our spatially explicit predictive model of crop damage only included land-use data without data on pest abundance, which allowed us to identify potential priority areas after the spatial arrangement of arable fields in a certain year has been determined. The predicted value for pecky rice damage was relatively stable for estimation of the hazard to brown rice first and second grades from arcsine-transformed values of zero to three (Fig. 1). However, the value was not sensitive enough for a quantitative prediction (Fig. 1), and the model prediction of more severe pecky rice damage was generally underestimated (Fig. 1a and c). This prediction tendency would be derived from the effect of research year (Additional file 1: Table S1), and the biological phenomenon caused by *S. rubrovittatus*. The sites with a lower percentage source habitat stably suffer lower damage, and more severe damage occurs unexpectedly at sites with a higher percentage of source habitat. However, the model performed accurately enough to predict whether the brown rice would be first grade, which is the most important concern for local farmers. Thus, our results will be applicable for local farmers and others in determining appropriate pest management policies and to support decision-making in several regions. This model and the priority area map will help to determine the areas that should receive insecticide applications and the number of applications that an area may require. Farmers usually need to optimize the allocation of resources necessary to mitigate crop damage by spraying insecticides at the most effective period because of limited time and labor. By using the current predictive model, they will be able to focus their labor in high priority areas and might be able to omit spraying insecticides for low priority areas, which may lead to savings in costs and labor.

## Conclusions

In summary, at least for the three regions examined in this study, a common single predictive model for predicting crop damage using land use data within an empirically determined spatial scale can be applied in independent regions in the agricultural landscapes. However, the model application in this study has the limitation that the extended model was not extrapolated to other new regions and its accuracy was not tested; therefore, further investigation will be needed. Agricultural landscapes are anthropogenically managed, especially in regions that grow grain intensively. Under such relatively well-managed environments, it is valuable to know how to extrapolate spatially explicit predictive models after empirically examining the environmental conditions among regions. For efficient regional pest management, we will need to incorporate the ecological understanding of this study to model pest systems. Here, crop damage is usually related to pest abundance, so it was examined as an extended biological response of pests to landscape factors. Biological responses include a wide variety of factors such as species occurrence, abundance, and breeding [3], and cover several ecosystem services such as pest suppression and pollination. Little is known about the inter-regional common relationships of such responses and few studies have examined the challenges of extrapolation, so further work is needed in this area. From the point of view of model application, several environmental hazards such as plant pathogens [30, 31], wildlife [32], vector-borne diseases [33], and alien invasive species [26, 34], have been dealt with in previous studies. It might be possible to develop early-warning or preventative guidelines by offering region-wide management options. Such trials will be done in the near future and the application of the model will be validated in further studies.

## Methods

### Study insect

*Stenotus rubrovittatus* is a major rice pest causing pecky rice in eastern Asian countries including Japan [9, 10]. Contamination of brown rice with pecky rice grains results in a lower rice grade and affects the market price. Under Japanese rice quality regulations, there are four grades of brown rice (first, second and third grades, and substandard) based on the percentage of pecky rice: first grade ≤ 0.1%, 0.1% < second ≤ 0.3%, 0.3% < third ≤ 0.7%, and 0.7% < substandard [35]. *Stenotus rubrovittatus* occurs mainly in habitats dominated by graminoid weeds [12]. The abundance of the species in rice paddy fields increases after the flowering stage of the rice plant, and peaks sharply to make the connection between adults/nymph with rice and graminoid weeds easier. Numbers then decline after a week and increase again approximately 2 weeks after the first peak [36]. Indeed, while *S. rubrovittatus* nymphs prefer graminoid weeds to rice [37] and rice is not a good food resource for development, the adults of this species are preferentially attracted by volatiles of flowering rice panicles [38].

As mentioned earlier, Tabuchi et al. [11] developed a predictive model of pecky rice damage using land use within a 300-m radius which is the effective spatial scale of *S. rubrovittatus*, using a single application of insecticide for stinkbugs and one rice variety, ‘Hitomebore’. The functional spatial scale demonstrated from independent studies with different locations was found to be between 300 [13] and 400 m [14]. We set the effective functional spatial scale as a 300-m radius from the focal rice paddy fields in this study, based on these independent studies. The major factor affecting pecky rice damage in the predictive model was the area of source habitat for *S. rubrovittatus*. The area of soybean fields was examined as an additional component of the predictive model, but this was only marginally significant. Soybean itself is not a host plant of *S. rubrovittatus*; however, soybean fields that are not well managed for weeds often have a high density of graminoid weeds. Moreover, the abundance of *S. rubrovittatus* (i. e. the number of adult males caught by synthetic sex pheromone-baited traps) was not an important model parameter according to model selection using the AIC [11].

### Study site

**Fig. 4:**
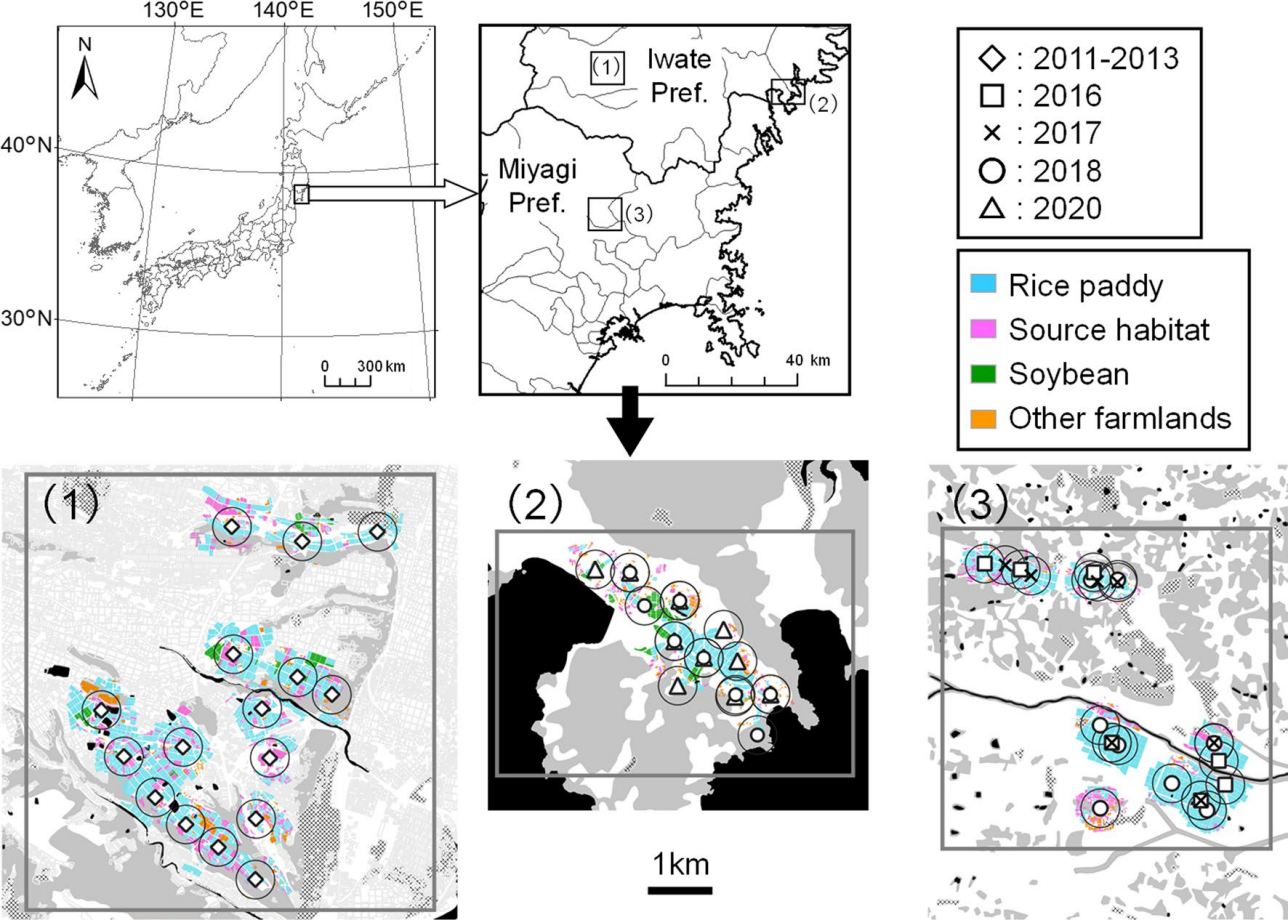
Map of the study area showing the positions of the rice paddy fields (white diamonds: previous study (Tabuchi et al. 2017); white squares, cross marks, circles and triangles: current study). Each circle centered on a point indicates a radius of 300 m. (1) Maesawa region, Oshu city, (2) Otomo region, Rikuzen-takata city, and (3) Semine region, Kurihara city. Agricultural land uses are indicated by different colors

The multi-year field study was conducted in two regions located in northern Honshu Island, Japan (Fig. 4, Table 3). Two-year (2018 and 2020) and 3-year (2016–2018) studies were performed for the Otomo and Semine regions, respectively. The data were compared with the data from the previous study for the Maesawa region from 2011 to 2013 [11]. Maesawa and Otomo regions were located in Iwate Prefecture, and Semine was located in Miyagi Prefecture. The distances from the Otomo and Semine regions to Maesawa were 51.8 km and 44.6 km, respectively. The study regions were agricultural landscapes dominated by rice paddy fields, with some forests, and other land uses such as crop fields other than rice, open water, and urban areas (Fig. 4, Additional file 1: Fig. S1). In Maesawa and Semine, which were located inland, the other land uses were mainly composed of pastures, fallow fields, and urban areas (Table 4). Otomo was located in a coastal area, and forests and rice paddy fields are the major components of the agricultural landscape with small soybean fields, fallow fields and residential areas. Maesawa and Semine were typical of the flat plain agricultural landscapes of northern Honshu Island, and Otomo was typical of agricultural landscapes in the coastal area of northern Honshu Island. In Maesawa, almost 90% of pastures contained Italian ryegrass, *Lolium multiflorum* Lam. (Poales: Poaceae), which is one of the major host plants for *S. rubrovittatus* (Nagasawa and Higuchi 2012). In Semine, pastures contained a mixture of Italian ryegrass and orchard grass *Dactylis glomerata* L. (Poales: Poaceae). Wild gramineous weeds, such as *Digitaria ciliaris* (Retz.) Koeler, *Setaria viridis* (L.) P.Beauv., *Echinochloa crus-galli* (L.) P.Beauv., *Imperata cylindrica* (L.) Raeusch., and *Polypogon fugax* Nees ex Steud., were observed in fallow fields. In Otomo, there were no pasture fields but several wild gramineous weeds such as *D. ciliaris*, *S. viridis*, *E. crus-galli*, and *I. cylindrica* were observed in fallow fields. The average area of source habitats, such as pastures that mainly consisted of Italian ryegrass and graminoid-dominated fallow fields was 11.0%, ranging from 1.3 to 29.4%, among the regions (Table 3, Additional file 1: Fig. S1). The present study sites were situated in a temperate zone. The mean temperature of the three regions over the research period from July to September was 20.3 °C, and mean precipitation per month during field research was 149.1 mm (Table 3).

**Table 3:** Summary of research locations

Location (Region, City, Prefecture)	Latitude	Longitude	Year	Total no. of rice fields examined	Mean temperature (°C)	Mean precipitation (mm/month)	Elevation (m)
Maesawa, Oshu, Iwate^a^	39.07267	141.10849	2011–2013	47	20.3	145.1	87.9
Otomo, Rikuzentakata, Iwate	38.99107	141.69709	2018 and 2020	18	20.1	142.6	10.8
Semine, Kurihara, Miyagi	38.65049	141.05932	2016–2018	28	20.3	157.3	9.0

Latitude and longitude are centroids of research fields in each location (datum: WGS84)

Mean temperature and precipitation averaged throughout the rice-growing season from May to September across all research years of each region

Meteorological data were obtained from the Japan Meteorological Agency (https://www.data.jma.go.jp/obd/stats/etrn/index.php)

Elevation is based on the digital elevation model of the 5-m mesh (DEM5a) data provided by the Geospatial Information Authority of Japan (https://maps.gsi.go.jp/)

^a^Data from Tabuchi et al. (2017)

^b^Data of current study

**Table 4:** Summary of fields examined in different regions

Location (Region, City, Prefecture)	Average area of rice paddy field examined ± SE (ha) (n)	Average area of fields investigated for land use in each year ± SE (ha) (n)	Average distance between fields ± SE (m)	Mean source habitat (%) (range)
Maesawa, Oshu, Iwate	0.22 ± 0.02 a (47)	0.15 ± 0.002 b (3508)	810.6 ± 27.4	14.65 (3.55–28.68)
Otomo, Rikuzen-Takata, Iwate	0.23 ± 0.02 a (18)	0.11 ± 0.003 c (1223)	559.6 ± 16.1	0.32 (0.13–0.53)
Semine, Kurihara, Miyagi	0.28 ± 0.04 a (28)	0.16 ± 0.005 a (1911)	596.1 ± 89.8	9.34 (0.84–29.09)

Different lowercase letters in each column indicates a significant difference (*p* < 0.001) by one-way ANOVA with the Tukey–Kramer HSD test

### Experimental design

To examine the inter-regional generality of the relationship between pecky rice damage and land use, surveys of land-use types in the focal area were conducted every year during the multi-year field research periods for two regions, each in a different year for 4 years in total (Tables 3 and 4). The the data were compared with the previous data in the Maesawa region from 2011 to 2013 [11]. We categorized seven land-use types: forest, rice paddy fields, soybean fields, other agricultural fields, source habitats of *S. rubrovittatus* (such as pastures and graminoid-dominated fallow fields), open water, and other land uses (Table 4). We set a research point of 10 m from the corner of the focal rice paddy field to avoid any effects of *S. rubrovittatus* movement caused by the mowing of paddy field borders [36]. Within a 300-m radius around the focal field of each site, each land use type was identified using a combination of aerial photos taken by a commercial UAV (DJI Mavic Pro and DJI Mavic 2 Pro, DJI, Shenzhen, China) and visual field assessment (i.e., ground-truthing), for a total of 6,642 fields. The data were mapped and the area of each land use calculated using ArcGIS 10.4 [39]. We used the ArcGIS license of AFFRIT, MAFF, Japan, in this research. An arable field polygon dataset was provided by the Federation of Land Improvement Associations of Iwate Prefecture and Miyagi Prefecture, and the Ministry of Agriculture, Forestry and Fisheries [40]. The arable field polygon dataset only included arable land parcel information with no information available on planted crops.

Most agricultural field margins were dominated by graminoids, suggesting that each field margin could act as a potential source habitat as previously reported (e.g. [13]). We calculated the area of field margin surrounding each focal field using an equation based on the agricultural field area (m^2^) (the area of field margin = 0.3978 × field perimeter (m) − 25.173), where 0.3978 is the coefficient and − 25.173 is the intercept (Additional file 1: Fig. S4). A highly positive relationship was shown between the area of field margin and the field perimeter (*R*^2^ = 0.602, *n* = 33, *p* < 0.001). The calculated area of the field margin was added as one of the source habitats.

The three regions examined were fine-grained agricultural landscapes, and the average size (± SE) of a rice paddy field examined was 0.24 ± 0.02 ha (Table 4). The average area (± SE) of fields investigated fir land use at Maesawa, Otomo, and Semine was 0.15 ± 0.002, 0.10 ± 0.003, and 0.16 ± 0.004 ha, respectively, and significantly differed among regions (one-way Anova, *F*_2,6639_ = 37.86, *p* < 0.001, Additional file 1: Fig. S5). Therefore, there was an effect of land use configuration to some extent; however, most fields were in the interquartile range from 0 to 0.4 ha and overlapped, so that the effect was not likely to be serious among regions in this study. The average distance (± SE) between traps set in rice paddy fields was 809 ± 49 m, ranging from 596 to 1179 m (Table 4). All rice paddy fields were managed conventionally by local growers, and the rice varieties ‘Hitomebore’ in Maesawa and Otomo and ‘Sasa-nishiki’ in Semine was grown from mid-May to mid-October. Insecticide input for all research fields was controlled, and an insecticide (dinotefuran) was sprayed once for pests attacking rice grains in early August in every research year by a radio-controlled helicopter. Another insecticide (chlorantraniliprole) was applied to all fields examined in mid-May, just before transplantation of rice seedlings by nursery-box application.

We checked the degree of hull-cracked rice grain occurrence (Additional file 1: Fig. S6), which influences pecky rice damage and depends on each rice variety. The level of pecky rice damage caused by *S. rubrovittatus* and the number of hull-cracked rice grains were common among the rice varieties [41], and the degree of damage could be estimated by using the degree of hull-cracked rice grains regardless of the rice variety. However, the percentage of hull-cracked rice grains among the current study regions did not exceed that of the original region of the model, thus we did not need to correct the damage level among regions.

We selected focal paddy fields in this study that contained no graminoid weeds, such as *Echinochloa* spp. (Poales: Poaceae), or Cyperaceae weeds. These plants are suitable host plants for *S. rubrovittatus*, and enhance pecky rice damage [42].

To confirm whether *S. rubrovittatus* actually caused pecky rice damage, and to investigate the species composition of heteropteran pests causing pecky rice damage, we conducted sweeping net surveys at selected rice paddy fields (Additional file 1: Table S3). Among the heteropterans sampled that can cause pecky rice damage within the study regions, 80.4% (range 46.7–98.0%) of the individuals were *S. rubrovittatus*.

The pecky rice damage was investigated by collecting plants from 20 rice hills containing 32,131 rice grains on average (range 18,498–46,049 grains) from each rice paddy in every year. After hulling, brown rice grains were sifted through a 1.9 mm mesh. We counted the number of intact and damaged grains and calculated the degree of rice damage.

### Data analyses and model evaluation

All analyses were conducted using R 4.0.0 [43]. The predictive model of pecky rice damage [11] is shown as the following equation:

3 $$y = \, - 0.0{9} + { 4}0.0{5}*{\text{Source habitat }} + { 52}.{7}*{\text{Soybean }} - { 1}.{12}*{\text{Paddy field }} + {\text{ RI}}_{{{\text{year}}}}$$

where *y* is arcsine-transformed pecky rice damage at given points and − 0.09 is a fixed intercept of the model. The areas of source habitat, soybean fields, and paddy fields in Eq. (3) were the data calculated within a 300-m radius of research points (km^2^), and values to the left of asterisks are coefficients of the model. These are the fixed parts of the model. In Eq. (3), RI year is the year-specific random intercept described by year-specific variance with a normal distribution, and is the random part of the model. The yearly random intercepts were larger in the order of 2011, 2012, and 2013.

Before constructing the extended predictive model, we examined the parallel slopes of the regression lines for the regions and years by One-Way Multivariate Analysis of Covariance to check whether we could combine data from the three regions for the four research years of the current study and 3 years of the previous study [11]. The arcsine-transformed percentage of pecky rice was examined as a response variable, and the area of land uses (source habitat, soybean field, and rice paddy field), which were important factors in the original predictive model of rice damage [11]), region, research year, and their two-way interactions were analyzed as fixed factors.

The percentage of pecky rice damage was analyzed using a general linear mixed model in the nlme package [44]. The percentage of pecky rice damage was arcsine transformed, and a Gaussian error distribution was applied. In the model, the area (km^2^) of soybean fields and rice paddy fields, and source habitat of *S. rubrovittatus*, which included pastures and graminoid-dominated fallow fields, were set as fixed factors. To construct a single common predictive model applicable in different regions and years, regions and research years were treated as two levels of random factors in the model: intercepts were calculated for the three regions and each research year in each region, so that the model had three region-specific random intercepts and eight year-specific random intercepts [three for Maesawa (2011–2013), three for Semine (2016–2018), and two for Otomo (2018 and 2020)]. Meanwhile, to find a better approach for model improvement, we also tried to apply the following models: the random slope model, the model with nested years across the region, and the Bayesian model. However, substantial improvement was not demonstrated in *R*^*2*^ and AIC values, so we used the extended predictive model (Eq. 1).

As mentioned above, the predictive model of pecky rice damage [11] is only composed of land use variables. We assigned values that were obtained from the current study to the original model and calculated the predicted value of pecky rice damage. In Tabuchi et al. [11], the random intercept was assigned the highest value in 2011 to obtain a conservative output. After that, to adjust the coefficients of each fixed factor to the data of the current study regions, we prepared an extended predictive model. Using data from the current study and the original study [11], we analyzed the pecky rice damage and prepared the model. The area of source habitat, soybean fields, and rice paddy fields within 300 m of the sampled field were set as fixed factors, and research year and regions were set as random factors. We ranked the plausibility of the models using Akaike’s information criterion adjusted for small sample sizes (AICc). If the difference in the ΔAICc was less than 2, then that model was considered more relevant than the other models [45]. Multicollinearity of the model (i.e. whether the variance inflation factor was less than 10; [46]) was also checked. When the extended model was statistically significant, we calculated the predicted value of pecky rice damage.

To evaluate the suitability of the extrapolation of the original model and whether the predicted values from the extended model improved on the original model, post hoc comparisons of the observed and predicted values calculated by the models were performed with a general linear mixed model using the nlme package. We compared both the data of the current study only, and the current and the original data combined. Research year and region were added to the model as random factors. The *R*^2^ values for the mixed models were calculated using the function r.squaredGLMM from the package MuMIn [47]. The predictive performance of the extended model for unknown data was evaluated by three-fold cross validation.

The accuracies of the original and the extended models for predicting the amount of first grade brown rice, which is of greatest importance to local farmers were measured and compared by the AUC curve [48] using observed and predicted values in the R package ROCR [49]. For the original and the extended models, positive and negative classification errors, and the proportion of correct predictions (sensitivity, specificity, and positive and negative predictive values), were calculated to determine the relevance of the model.

To demonstrate the sensitivity of the extended model to show how the prediction values of the model behave, we calculated the model prediction values for the lowest and highest risk cases, which were determined from the lowest and highest random intercepts. The predicted values were fitted using the minimum, average, and maximum areas of source habitat and soybean fields in the data, which were shown to be significant factors in the original predictive model in Tabuchi et al. [11]. In this calculation, the average area of rice paddy fields was used in all cases.

### Priority area map construction

On the basis of a predictive spatial model, we constructed a map showing the potential priority areas for pecky rice damage. Because we only investigated the surrounding land use of fields within 300 m radii of research points, for each region and surrounding wider area (Fig. 4) other than the research fields, we determined the land use of each field by visualization. Satellite and aerial imagery taken from DigitalGlobe, which was available as a base layer in ArcGIS 10.4 [39] in June 2012 for the Maesawa region and in August 2017 for the Semine region was used as a reference. For Otomo, we used the satellite imagery from Google Earth taken in October 2017. The results of direct observations of land-use types were used as a reference. In total, 18,319 agricultural fields and other land-use areas were determined and mapped. To construct the priority area map, the focal agricultural area was divided into hexagons with each side being 300 m, which is the maximum size fitting inside a circle with a 300-m radius. Only hexagons that included agricultural fields were selected. To rank the risk to rice paddy fields in each hexagon, the area of each land use type within each hexagon was calculated, and these data approximated a circle of 600-m diameter. The area of land use in each hexagon was assigned to the model for extrapolation to the surrounding area. The predicted values of pecky rice damage were classified according to the four brown rice grades and then mapped using color-coordinated hexagons.

## Supplementary Information

**Additional file 1. Table S1.** Summary of One way Multivariate Analysis of covariance model that examined the effects of the area of land uses (source habitat, soybea n field, and rice paddy field) within a 300 m radius, regions examined, and research years on arcsine transforme d pecky rice damage. **Table S2.** Summary of the model coefficients ((± 95% CI) of fixed factors and t he value of the fixed intercept for the extended model’ and the original model’ (Tabuchi et al., 2017). **Table S3.** Summary of net sweeping surveys in selected rice paddy fields. **Figure S1.** Percentage of the area of (**a**) source habitat, (**b**) soybean fields and (**c**) rice paddy fields within a 300-m radius in each region and year. The data are represented as box plots with median values as thick lines and mean values as diamonds, showing the 25th and 75th percentiles. Whiskers extend to the most extreme data point that is no more than 1.5 times the interquartile range from the box. Outliers are shown as open circles. **Figure S2.** Arcsine-transformed percentage of pecky rice damage in each region and year. The data are represented as box plots with median values as thick lines and mean values as diamonds, showing the 25th and 75th percentiles. Whiskers extend to the most extreme data point that is no more than 1.5 times the interquartile range from the box. Outliers are shown as circles. **Figure S3.** Priority area map of potential pecky rice damage of the highest risk (upper) and the lowest risk (bottom) case with a grid layer of 300 m hexagons. Shapes in the figure indicate the studied rice paddy fields. Regions: (1) Maesawa, (2) Otomo, and (3) Semine. **Figure S4.** Relationship between field perimeter (m) and the area of field margin. **Figure S5.** Area of rice fields examined (**a**) and the fields investigated for land use in each region (**b**). The data are represented as box plots with median values as thick lines and mean values as diamonds, showing the 25th and 75th percentiles. Whiskers extend to the most extreme data point that is no more than 1.5 times the interquartile range from the box. Outliers are shown as open circles. Different letters above the boxes indicate a significant difference (*p* < 0.001) by one-way ANOVA with the Tukey–Kramer HSD test. **Figure S6.** Percentage of hull-cracked rice grains in each region and year. The data are represented as box plots with median values as thick lines and mean values as diamonds, showing the 25th and 75th percentiles. Whiskers extend to the most extreme data point that is no more than 1.5 times the interquartile range from the box. Outliers are shown as open circles.

## Data Availability

The datasets generated during and/or analyzed during the current study are available in the GitHub Repository https://github.com/KTab6464/DamagePrediction_data_2021
