# Supplementary material for: Extrapolating potential crop damage by insect pests based on land use data: examining inter-regional generality in agricultural landscapes
Source: BMC Ecol Evol. 2022 May 25;22:70. doi: 10.1186/s12862-022-02024-7 (PMC9131665; doi:10.1186/s12862-022-02024-7)
Supplement: Supplementary file 1 — Additional file 1. Table S1. Summary of One way Multivariate Analysis of covariance model that examined the effects of the area of land uses (source habitat, soybea n field, and rice paddy field) within a 300 m radius, regions examined, and research years on arcsine transforme d pecky rice damage. Table S2. Summary of the model coefficients ((± 95% CI) of fixed factors and t he value of the fixed intercept for the extended model’ and the original model’ (Tabuchi et al., 2017). Table S3. Summary of net sweeping surveys in selected rice paddy fields. Figure S1. Percentage of the area of (a) source habitat, (b) soybean fields and (c) rice paddy fields within a 300-m radius in each region and year. The data are represented as box plots with median values as thick lines and mean values as diamonds, showing the 25th and 75th percentiles. Whiskers extend to the most extreme data point that is no more than 1.5 times the interquartile range from the box. Outliers are shown as open circles. Figure S2. Arcsine-transformed percentage of pecky rice damage in each region and year. The data are represented as box plots with median values as thick lines and mean values as diamonds, showing the 25th and 75th percentiles. Whiskers extend to the most extreme data point that is no more than 1.5 times the interquartile range from the box. Outliers are shown as circles. Figure S3. Priority area map of potential pecky rice damage of the highest risk (upper) and the lowest risk (bottom) case with a grid layer of 300 m hexagons. Shapes in the figure indicate the studied rice paddy fields. Regions: (1) Maesawa, (2) Otomo, and (3) Semine. Figure S4. Relationship between field perimeter (m) and the area of field margin. Figure S5. Area of rice fields examined (a) and the fields investigated for land use in each region (b). The data are represented as box plots with median values as thick lines and mean values as diamonds, showing the 25th and 75th percentiles. Whiskers extend to the most extre [file 12862_2022_2024_MOESM1_ESM.doc]

Additional file for

**Extrapolating potential crop damage by insect pests based on land use data: examining inter-regional generality in agricultural landscapes**

Authors: Ken Tabuchi ^1^, Akihiko Takahashi ^1, 2^, Ryuji Uesugi ^1^, Shigeru Okudera ^1, 3^, and Hideto Yoshimura ^1^

^1^ Tohoku Agricultural Research Center, NARO, 4 Akahira, Shimo-kuriyagawa, Morioka, Iwate 020-0198, Japan

^2^ Hokuriku Research Station, Central Region Agricultural Research Center, NARO, 1-2-1 Inada, Joetsu, Niigata 943-0193, Japan

^3^ Laboratory of Biology, Asahikawa Campus, Hokkaido University of Education, 9 Hokumon-cho, Asahikawa 070-8621, Japan

Table S1 Summary of One-way Multivariate Analysis of covariance model that examined the effects of the area of land uses (source habitat, soybean field, and rice paddy field) within a 300-m radius, regions examined, and research years on arcsine-transformed pecky rice damage

|  | SS | df | *F* | *P* |
| --- | --- | --- | --- | --- |
| Source habitat | 21.44 | 1 | 16.86 | < **0.001** *** |
| Soybean300 | 8.518 | 1 | 6.70 | **0.011 *** |
| Rice paddy | 0.806 | 1 | 0.63 | 0.429 |
| Region | 0.091 | 1 | 0.07 | 0.789 |
| Year | 27.283 | 5 | 4.29 | **0.002 **** |
| Source habitat * Region | 0.878 | 1 | 0.69 | 0.409 |
| Soybean * Region | – | 0 | – | – |
| Rice paddy * Region | 0.026 | 1 | 0.0207 | 0.886 |
| Source habitat * Year | 15.214 | 5 | 2.3931 | **0.047 *** |
| Soybean * Year | 0.212 | 3 | 0.0555 | 0.983 |
| Rice paddy * Year | 0.56 | 5 | 0.0882 | 0.994 |
| Residuals | 81.375 | 64 |  |  |

*: < 0.05, **: < 0.01, ***: <0.001. Values without * are not significant

Bold characters indicate significant variables

Table S2 Summary of the model coefficients (± 95% CI) of fixed factors and the value of the fixed intercept for the ‘extended model’ and the ‘original model’ (Tabuchi et al., 2017)

|  | Value and coefficients  (± 95% CI) | |
| --- | --- | --- |
|  | ‘Extended’ model  (current study, equation (1)) | ‘Original’ model  (Tabuchi et al., 2017, equation (2)) |
| Source habitat | **32.071 *****  (17.22–46.92) | **40.050 *****  (16.71–63.39) |
| Soybean | **44.515 ***  (9.99–79.04) | 52.699 †  (-3.03–108.43) |
| Paddy field | 2.614  (-2.52–7.75) | -1.118  (-13.72–11.49) |
| Fixed Intercept | 0.032  (-0.77–0.83) | -0.088  (-1.55–1.38) |

†: < 0.10*: < 0.05, **: < 0.01, ***: <0.001. Values without * are not significant

Each value of the model was calculated with the function “Confint ()” of the car package in R

Bold characters indicate significant variables

Table S3 Summary of net sweeping surveys in selected rice paddy fields ^1)^

| Location  (Region, City, Prefecture) | Year | No. of fields | % of *Stenotus rubrovittatus* | Total | *S. rubrovittatus* | *Trigonotylus caelestialium* | *Stenodema calcarata* | *Adelphocoris lineolatus* | *Rhopalus maculatus* | *Cletus punctiger* | *Leptocorisa chinensis* | *Eysarcoris aenus* |
| --- | --- | --- | --- | --- | --- | --- | --- | --- | --- | --- | --- | --- |
| Otomo, Rikuzen-Takata,  Iwate | 2019^2)^ | 9 | 82.5 | 268 | 221 | 0 | 12 | 0 | 0 | 0 | 33 | 2 |
|  | 2020 | 10 | 46.7 | 15 | 7 | 1 | 2 | 0 | 0 | 0 | 3 | 2 |
| Semine, Kurihara,  Miyagi | 2016 | 2 | 96.3 | 54 | 52 | 1 | 0 | 0 | 0 | 1 | 0 | 0 |
|  | 2017 | 2 | 98.0 | 149 | 146 | 2 | 1 | 0 | 0 | 0 | 0 | 0 |
|  | 2018 | 3 | 78.4 | 97 | 76 | 19 | 1 | 0 | 0 | 0 | 0 | 1 |

1) A net sweeping survey was conducted weekly from mid-July to early September in the Maesawa and Semine regions. We set two transects about 10m long beside a point of 10 m from the corner of the paddy field and swept 20 times for each transect (i.e. total 40 sweeps) using a 36-cm diameter sweeping net with 90cm long handle, which is a standard protocol recommended by Japanese law to investigate the abundance of rice pests

2) In the Otomo region, we did not conduct a net sweeping survey in 2018. However, three net sweeping surveys were conducted in nine rice paddy fields during early August to early September in 2019 to collect reference data

Figure S1 Percentage of the area of (a) source habitat, (b) soybean fields and (c) rice paddy fields within a 300-m radius in each region and year. The data are represented as box plots with median values as thick lines and mean values as diamonds, showing the 25th and 75th percentiles. Whiskers extend to the most extreme data point that is no more than 1.5 times the interquartile range from the box. Outliers are shown as open circles.

Figure S2 Arcsine-transformed percentage of pecky rice damage in each region and year. The data are represented as box plots with median values as thick lines and mean values as diamonds, showing the 25th and 75th percentiles. Whiskers extend to the most extreme data point that is no more than 1.5 times the interquartile range from the box. Outliers are shown as circles.

Figure S3 Priority area map of potential pecky rice damage of the highest risk (upper) and the lowest risk (bottom) case with a grid layer of 300-m hexagons. Shapes in the figure indicate the studied rice paddy fields. Regions: (1) Maesawa, (2) Otomo, and (3) Semine.


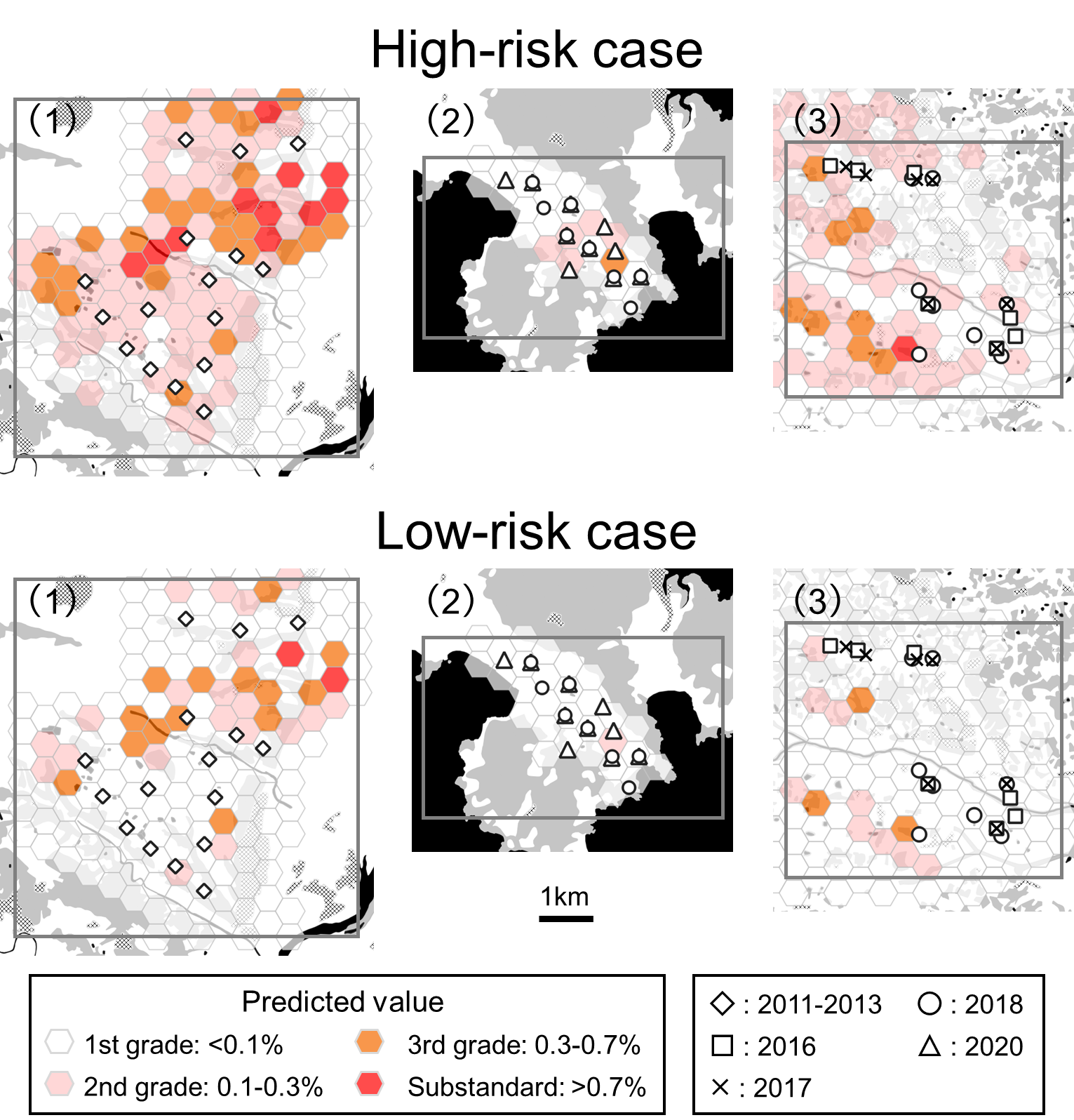


Figure S4 Relationship between field perimeter (m) and the area of field margin.

Figure S5 Area of rice fields examined (a) and the fields investigated for land use in each region (b). The data are represented as box plots with median values as thick lines and mean values as diamonds, showing the 25th and 75th percentiles. Whiskers extend to the most extreme data point that is no more than 1.5 times the interquartile range from the box. Outliers are shown as open circles. Different letters above the boxes indicate a significant difference (*p* < 0.001) by one-way ANOVA with the Tukey–Kramer HSD test.

Figure S6 Percentage of hull-cracked rice grains in each region and year. The data are represented as box plots with median values as thick lines and mean values as diamonds, showing the 25th and 75th percentiles. Whiskers extend to the most extreme data point that is no more than 1.5 times the interquartile range from the box. Outliers are shown as open circles.
